# Signaling pathways of marine-derived natural products on lung injury: from the *in vivo* perspective

**DOI:** 10.3389/fphar.2025.1716898

**Published:** 2026-01-05

**Authors:** Haitong Wang, Feng Qiu

**Affiliations:** 1 The First Affiliated Hospital, Dalian Medical University, Dalian, China; 2 College of Pharmacy, Dalian Medical University, Dalian, China

**Keywords:** *in vivo*, inflammatory response, lung injury, marine natural products, signaling pathway

## Abstract

Lung injury is characterized by impaired gas exchange, inflammatory responses, and abnormal tissue repair. In severe cases, it can progress to respiratory failure, posing a threat to public health. Marine natural products, due to their structural and biological activity diversity, show significant potential in the treatment of lung injury. This article systematically reviews the mechanism by which marine-derived natural products improve lung injury by regulating key signaling pathways *in vivo*. Studies have demonstrated that marine natural products target pathological processes such as inflammatory immunity and oxidative stress by regulating signaling pathways including αvβ3-FAK/Src, TLR4/MyD88, NF-κB, and Keap1-Nrf2/HO-1/STAT3, thereby exerting a significant protective effect on alveolar structures in lung injury models induced by stimuli such as radiation, OVA, LPS, and cigarette smoke. Currently, there is a lack of marine drugs specifically for lung injury, and in-depth research is needed to promote their translation into clinical medications.

## 1 Introduction

**FIGURE 1:**
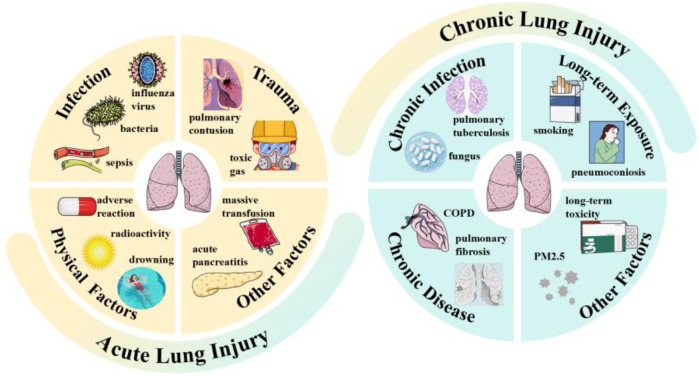
Types and inducing factors of lung injury.

The lung, as the most vital organ in the human respiratory system, facilitates gas exchange with inhaled air to acquire the oxygen requisite for cellular respiration (Luo et al., 2025). Lung injury refers to the pathophysiological process characterized by impaired gas exchange and inflammatory responses, which is triggered by various damaging factors that induce pulmonary tissue damage, with clinical manifestations of dyspnea (Dubey et al., 2025), hypoxemia (Swenson et al., 2021), cough and expectoration (Guo J. et al., 2024), in severe cases, it can progress to respiratory failure (Fedt et al., 2020). Its pathological core involves increased pulmonary microvascular permeability (Knudsen and Ochs, 2018), alveolar epithelial injury (Swenson et al., 2021), inflammatory cell infiltration (Ma L. et al., 2025), and abnormal tissue repair (D'Agnillo et al., 2021), etc. Based on clinical characteristics, it can be classified into two types: acute injury and chronic injury, as illustrated in Figure 1.

Among them, acute lung injury is associated with high morbidity and mortality, and can progress to acute respiratory distress syndrome with poor prognosis, with a mortality rate as high as 40% (Liao et al., 2021). During the early stages of the COVID-19 pandemic and the 2002–2003 severe acute respiratory syndrome regional outbreak, the mortality rates of ICU patients who died from acute respiratory distress syndrome reached as high as 67%–85% and 52.2%, respectively (Zhang et al., 2024). According to recent statistical data released by the World Health Organization, it is estimated that due to exposure to air environmental pollution, infections, or genetic backgrounds, the global population will develop acute or chronic lung diseases within the next decade (Bezerra et al., 2023). Even with the rapid advancements in current medical technologies, lung injury-related diseases still pose a significant public health threat.

In recent decades, the potential of marine natural products in the medical field has gained increasing recognition, characterized by extensive chemical activities and biological diversity. Marine microorganisms, algae, and invertebrates constitute the three primary sources of marine natural products (Zhang et al., 2025; Bezerra et al., 2023). These compounds are involved in key *in vivo* processes such as tumor cell cycle regulation, autophagy, ferroptosis, and gut microbiota programming, exhibiting potent anti-inflammatory and anti-tumor potentials. Furthermore, marine natural products have demonstrated significant antiviral, antibacterial, and antifungal activities (Ma M. T. et al., 2025). Such remarkable efficacy may be attributed to their unique functional groups (e.g., brominated and chlorinated alkaloids), complex cyclic structures (e.g., macrolides and polyether compounds), and the presence of sulfate groups (Zhang et al., 2017).

The application of marine natural products in the treatment of lung injury holds theoretical feasibility. From the perspective of pathological mechanisms, the multi-target regulatory properties of marine natural products enable them to specifically address pathological processes associated with lung injury, such as excessive inflammatory responses, oxidative stress damage, and pulmonary fibrosis (Kumar and Adki, 2018). For instance, the carotenoid fucoxanthin can inhibit the production of inflammatory cytokines triggered by *Mycoplasma* pneumoniae and enhance bacterial clearance in mouse models (Li et al., 2020); astaxanthin can reduce the increase in inflammatory cells in bronchoalveolar lavage fluid and emphysema (Akduman et al., 2021; Bi et al., 2017); and fucoidan significantly alleviates hyperoxia-induced lung injury (Nie et al., 2018). To date, a large number of natural compounds with lung injury-protective activity have been isolated from marine microorganisms, algae, sponges, and other organisms, including alkaloids, terpenoids, polypeptides, polyketides, and pyranones (Zhang Y. et al., 2020; Singh, 2020). However, current research has obvious limitations. On one hand, most studies remain in the stages of compound isolation, identification, and *in vitro* activity screening, verifying their protective effects on lung epithelial cells, macrophages, etc., through cell experiments or extracellular enzyme experiments, while in-depth *in vivo* studies are relatively scarce. The *in vivo* environment is a complex dynamic system involving interactions among various cells, tissues, and physiological processes. On the other hand, the specific molecular mechanisms and targets underlying their therapeutic effects *in vivo*, especially the regulatory mechanisms of signaling pathways, remain unclear. Therefore, systematically sorting out the mechanistic pathways of marine natural products in the *in vivo* treatment of lung injury and clarifying the current problems and challenges are of great significance for developing new therapeutic strategies for lung injury and accelerating the development and application of marine natural products.

## 2 Molecular mechanisms/pathways of lung injury for marine natural products

### 2.1 Immune response and inflammation

The development of lung injury is closely associated with abnormal activation of the immune system and the inflammatory response. When the lungs encounter external stimuli such as radiation, allergens, pathogens, or chemicals, the innate and adaptive immune systems become activated. This activation leads to a substantial release of pro-inflammatory cytokines, including TNF-α, IL-1β, and IL-6. Additionally, inflammatory cells such as neutrophils, macrophages, and T-cells infiltrate the lung tissue. This process triggers oxidative stress, tissue edema, and damage to the alveolar structure. These immune-inflammatory responses form a common pathological basis for various types of lung injuries. Currently, several *in vivo* models are used to study the mechanisms behind lung injury due to immune-inflammatory responses. These models include radiation-induced lung injury, OVA-induced T cell activation, lipopolysaccharide (LPS)-induced lung injury, and cigarette smoke-induced lung injury models. Research has shown that a range of marine natural products exhibited significant protective effects in these four models.

#### 2.1.1 Marine natural products for radiation-induced lung injury

Radiation-induced lung injury (RILI) is a common complication that occurs after radiotherapy for thoracic tumors. It is characterized by early-stage alveolar inflammatory exudation, infiltration of immune cells (such as macrophages and neutrophils), and the release of pro-inflammatory cytokines like IL-6 and TNF-α. In the late stage, RILI features abnormal activation of fibroblasts, deposition of extracellular matrix (ECM), and irreversible remodeling of lung tissue structure (Arroyo-Hernández et al., 2021). Several immune-related signaling pathways play a role in RILI. One significant pathway is the TGF-β/Smad pathway, which, when activated by radiation, triggers fibroblast activation, ECM deposition, and epithelial-mesenchymal transition, ultimately leading to irreversible fibrosis in lung tissue (Cao et al., 2017; Park et al., 2015). In addition, the HMGB1/TLR4 pathway serves as a central mechanism driving inflammation in RILI. This pathway amplifies the inflammatory response by activating multiple downstream pathways, including NF-κB and MAPK (Zheng et al., 2020). Research has shown that astaxanthin, a compound commonly found in shrimp, oysters, and other marine organisms, possesses strong anti-inflammatory and antioxidant properties. In mouse models of RILI, astaxanthin has been shown to inhibit the progression of pulmonary fibrosis and reduce levels of inflammatory factors. Furthermore, astaxanthin can alleviate RILI by inhibiting cell apoptosis *in vitro* (Li et al., 2023). As a natural marine product, astaxanthin is highly safe and widely sourced, making it a suitable candidate for adjuvant treatment of RILI.

#### 2.1.2 Marine natural products for OVA-induced T cell activation

Ovalbumin (OVA) is the primary protein found in egg white and serves as an exogenous protein that activates the mammalian immune system. It triggers T cell activation and induces a Th2-type immune response, mimicking the immune mechanisms involved in human allergic asthma (Azman et al., 2021). During OVA-induced T cell activation, various immune pathways related to antigen recognition, T cell activation, differentiation, and effector functions are engaged. The T cell receptor pathway is key for antigen recognition and activation (Shin et al., 2021). When CD28 on the surface of T cells binds to B7 on antigen-presenting cells, it enhances the intensity of T cell receptor signaling (Linsley et al., 1993). Additionally, CD28 recruits PI3K, which activates the Akt/mTOR pathway (Efimova and Kelley, 2009). The MAPK pathway is involved in regulating T cell proliferation and differentiation (Jiang et al., 2023). Further, the TGF-β-Smad pathway influences the direction of T-cell differentiation (Liu et al., 2025). In conclusion, OVA-induced T-cell activation results from the synergistic action of multiple pathways. Marine microorganisms produce secondary metabolites with unique structural and therapeutic potential. One such metabolite is alternariol, derived from the marine fungus Alternaria sp. Alternariol has been found to target early T-cell activation, impact T-cell apoptosis, and inhibit T-cell proliferation and inflammatory factor production. Notably, alternariol has demonstrated the ability to alleviate OVA-induced inflammation in mouse lungs by inhibiting T cell activation and preventing T cell metastasis to mouse lungs (Liu et al., 2025). The inhibitory effect of alternariol on OVA-induced T cell activation underscores the potential of marine microbial resources for drug discovery. Blocking T cell activation and migration is one therapeutic strategy for intervening in allergic asthma (Zhang et al., 2022). However, it is essential to focus on specifically modulating pathogenic T cell subsets while preserving the protective immune response to ensure safer and more effective clinical applications.

#### 2.1.3 Marine natural products for LPS-induced lung injury

Acute lung injury (ALI) is a prevalent respiratory condition characterized by increased permeability of the alveolar-capillary membranes. It manifests as noncardiogenic pulmonary edema and severe hypoxemia. The more severe form of ALI is called acute respiratory distress syndrome (ARDS) (Long et al., 2022). A classic experimental model for studying ALI and ARDS is the LPS-induced lung injury model. LPS triggers inflammatory gene transcription and cellular stress responses through activating members of the MAPK family (p38, JNK, ERK) (Wang et al., 2018). The activation of the NLRP3 inflammasome further exacerbates inflammatory injury (Ning et al., 2020). Additionally, LPS-induced reactive oxygen species (ROS) production activates the NLRP3 inflammasome, amplifying the inflammatory response (Liu Y. et al., 2024). The LPS-induced lung injury model also involves the regulation of the JAK-STAT pathway (Hashimoto et al., 2020) and PI3K/Akt signaling pathway (Hu et al., 2020). Together, these mechanisms lead to disruption of the alveolar-capillary barrier, neutrophil infiltration, and the release of numerous proinflammatory factors, closely mimicking the pathologic features of clinical ALI/ARDS. In studies involving mice with LPS-induced acute lung injury, hypobranchial gland extract and the brominated indole compound 6-bromoisatin derived from the marine gastropod molluscs Dicathais orbita have been shown to inhibit inflammatory signaling pathways. These substances work by blocking the translocation of NF-κB to the nucleus and preventing macrophage activation (Ahmad et al., 2017). Furthermore, laminarin, a bioactive polysaccharide found in brown algae, can attenuate sepsis-associated ALI by decreasing M1 macrophage polarization through the downregulation of HIF-1α signaling (Zeng et al., 2025). Thus, marine natural products present significant potential for the treatment of ALI.

#### 2.1.4 Marine natural products for cigarette smoke-induced lung injury

Smoking is responsible for numerous lung diseases, particularly chronic obstructive pulmonary disease (COPD) and lung cancer (Durham and Adcock, 2015). The cigarette smoke-induced lung injury model effectively simulates COPD or emphysem Marine natural products for cigarette smoke-induced lung injury a that arises from long-term smoking or exposure to secondhand smoke. This model triggers chronic inflammation of the airways, destruction of alveolar structures, and excessive mucus production due to sustained exposure to smoke, similar to the pathological features of human smokers (Upadhyay et al., 2023). The immune mechanisms involved in this process include the activation of NLRP3 inflammasomes (Zhang et al., 2021), an imbalance between Th17 and regulatory T cells (Tao et al., 2025), and the polarization of macrophages into the M1 subtype (Feng and Zheng, 2023). In addition, oxidative stress exacerbates the inflammatory response by inhibiting the Nrf2 antioxidant pathway (Hikich et al., 2019), creating a vicious cycle of chronic lung injury. Collectively, these pathways reflect the immune-inflammatory cascade characteristic of smoking-related lung diseases. In the coastal region of southern China, a black coral extract from the genus Antipathes is being used to make cigarette holders that filter out some harmful substances in cigarette smoke (Bai et al., 2011a). Studies have shown that ultrasonically aerosolized inhalation of this extract can reduce cigarette smoke-induced inflammation in the lungs of mice with lung injuries, exhibiting both antioxidant and anti-inflammatory properties (Bai et al., 2011b). This black coral extract may have the potential as a protective agent against smoking-associated lung injuries, and nebulized inhalation could prove to be a more effective alternative to traditional oral formulations.

### 2.2 Keap1-Nrf2/HO-1/STAT3 pathway

#### 2.2.1 Keap1-Nrf2/HO-1/STAT3 signaling in lung injury

The mechanism of action of the Keap1-Nrf2/HO-1/STAT3 pathway in lung injury involves several key biological processes, including oxidative stress, inflammatory response, and cell survival. The respiratory system is particularly vulnerable to oxidative stress, making the Keap1-Nrf2 pathway essential for antioxidant defence (Abed et al., 2015). Under normal conditions, Keap1 functions as an adaptor protein for the E3 ubiquitin ligase complex, which binds to Nrf2, leading to its ubiquitination and subsequent degradation. This process keeps low levels of Nrf2. However, during lung injury, ROS induce a conformational change in Keap1, resulting in the release of Nrf2. Free Nrf2 then translocates to the nucleus, where it binds to the antioxidant response element and initiates the expression of downstream antioxidant genes, such as HO-1 and glutathione synthetase (Hikichi et al., 2019). HO-1, a crucial target gene for Nrf2, catalyzes the production of heme, which has antioxidant, anti-inflammatory, and iron metabolism properties (Ghareghomi et al., 2023). Additionally, in the context of lung injury, STAT3 also serves as a target gene for Nrf2 and plays a pivotal role in this pathway. STAT3 can be activated by cytokines or growth factors like IL-6 and IL-10. Once activated, it undergoes phosphorylation, forms a dimer, and moves into the nucleus, where it regulates genes related to inflammation, proliferation and apoptosis. Overactivated STAT3 may promote M1 macrophage polarization and the release of pro-inflammatory factors, intensifying acute inflammation or driving lung fibrosis. On the other hand, moderate activation of STAT3 supports alveolar epithelial cell proliferation, tissue repair, and cell survival through the upregulation of anti-apoptotic proteins such as Bcl-2 (Harada et al., 2014). In cases of acute lung injury activating the Keap1-Nrf2/HO-1 pathway can reduce oxidative damage, while controlling STAT3 activation may help alleviate inflammation.

#### 2.2.2 Marine-derived products in Keap1-Nrf2/HO-1/STAT3 pathway regulation

**FIGURE 2:**
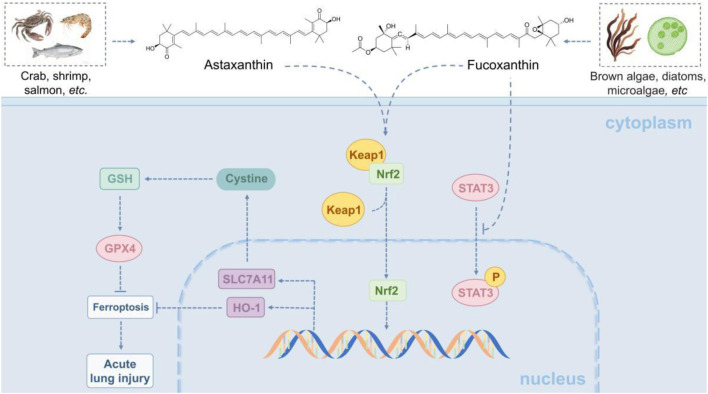
Marine-derived products attenuate acute lung injury (ALI) via the Keap1-Nrf2/HO-1/STAT3 pathway. The Keap1-Nrf2/HO-1/STAT3 pathway modulates oxidative stress, inflammation, and cell survival in lung injury. Normally, Keap1 degrades Nrf2; upon injury, ROS frees Nrf2, which enters the nucleus to activate HO-1 and other antioxidant genes. HO-1 has antioxidant, anti-inflammatory effects. STAT3, an Nrf2 target activated by IL-6/10, regulates related genes-overactivation worsens inflammation, while moderate activation promotes repair via Bcl-2. Marine astaxanthin and fucoxanthin protect against LPS-induced ALI: the former inhibits NF-κB and activates the pathway; the latter reduces lipid peroxidation and modulates Nrf2/STAT3. Both improve alveolar integrity.

Astaxanthin and fucoxanthin, two natural carotenoids derived from marine sources, both demonstrated significant anti-inflammatory effects and protection against iron-mediated cell death in a model of ALI induced by LPS. As astaxanthin was shown to reduce the expression of pro-inflammatory factors, such as COX-2 and iNOS, and to decrease NO release by inhibiting the nuclear translocation of NF-κB in acute and radio-induce lung injury (Luo et al., 2022). Additionally, it activated the Keap1-Nrf2/HO-1 pathway, which enhanced antioxidant capacity and reversed the abnormal expression of proteins related to iron death, including GPX4, SLC7A11, and FTH1, thus alleviating lung edema and inflammatory cell infiltration (Harada et al., 2014). Similarly, fucoxanthin significantly reduced lipid peroxidation levels and the accumulation of ferrous ions by inhibiting inflammatory markers such as PTGS2 and TNF-α. It also modulated the Nrf2/STAT3 pathway and the glutathione metabolism pathway, resulting in an upregulation of glutathione and its metabolic intermediates such as γ-Glu-Cys, to enhance antioxidant defence (Ding et al., 2024). Both compounds were effective in improving alveolar barrier integrity and mitigating pathological inflammatory responses by modulating Nrf2-associated antioxidant pathways and targeting key proteins involved in iron death. The mechanism by which they alleviated ALI through the Keap1-Nrf2/HO-1/STAT3 pathway is illustrated in Figure 2.

### 2.3 NF-κB-related pathways

#### 2.3.1 NF-κB-related pathways in lung injury

The nuclear factor κB (NF-κB) pathway is a crucial signaling pathway that regulates inflammation and immune response. It plays a significant role in ALI and chronic lung diseases (Millar et al., 2022). When stimulatory factors such as LPS, TNF-α, or IL-1β bind to their respective receptors, they activate the IKK complex. This activation leads to the degradation of the inhibitory protein IκBα and the subsequent release of NF-κB (p50/p65) into the nucleus, where it initiates the transcription of pro-inflammatory genes. In cases of lung injury, the overactivation of NF-κB leads to the upregulation of inflammatory factors. This recruitment of inflammatory mediators results in neutrophil infiltration, damage to the alveolar epithelial and endothelial barriers, and the onset of lung oedema (Li et al., 2022). Additionally, NF-κB promotes the expression of NADPH oxidase and ROS generation. This increase in ROS contributes to lipid peroxidation and DNA damage, thereby exacerbating lung injury (Harijith et al., 2022; Carnesecchi et al., 2011). Chronic activation of NF-κB further stimulates the secretion of factors like TGF-β and PDGF, which activate fibroblasts and can result in lung fibrosis (Liu and Desai, 2015). Moreover, the NF-κB pathway shares common upstream activators with the MAPK and PI3K/Akt pathways, and there exists a cross-regulation among these pathways, collectively influencing biological processes such as inflammation, cell survival, proliferation, and apoptosis (Guo Q. et al., 2024).

#### 2.3.2 Marine-derived products in NF-κB-related pathways regulation

**FIGURE 3:**
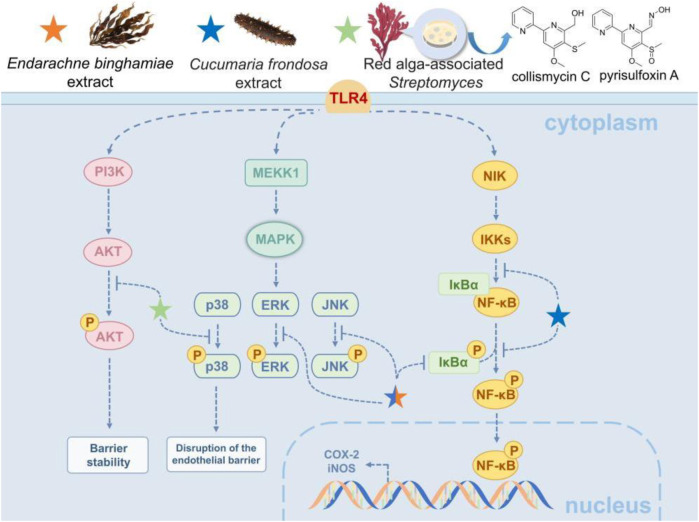
Marine-derived products attenuate ALI via NF-κB-related pathways. The NF-κB pathway, a core regulator of inflammation and immune response, is pivotal in ALI and chronic lung diseases. When LPS, TNF-α or IL-1β binds to receptors, it activates the IKK complex, triggering IκBα degradation and NF-κB nuclear translocation to transcribe pro-inflammatory genes, leading to lung edema and fibrosis. Marine natural products exert protective effects and alleviate injury by targeting NF-κB and related pathways.

Marine natural products offer multiple protective effects in inflammatory lung injury by targeting NF-κB and related signaling pathways. For instance, the extract of the marine brown alga Endarachne binghamiae significantly reduces pro-inflammatory factors like TNF-α, IL-6, and IL-1β in macrophages and lung tissues within an LPS-induced ALI model (Lee et al., 2025). This reduction occurs through the inhibition of TLR4-mediated NF-κB activation by blocking IκB phosphorylation and the MAPK (ERK/JNK/p38) pathway. The extract also decreases the expression of iNOS and COX-2 in macrophages and lung tissues, while reducing alveolar edema and inflammatory infiltration (Lee et al., 2025). Similarly, extract from the sea cucumber (Cucumaria frondosa) also lowers IL-1β and TNF-α levels by synergistically inhibiting the phosphorylation of the NF-κB/MAPK/JNK pathways in acute lung injury (Fagbohun et al., 2024). When combined with wild blueberry polyphenols, such as anthocyanins, they further modulate IL-4 expression, which helps TLR4-mediated damage to the alveolar barrier (Fagbohun et al., 2024). Furthermore, 2, 2′-bipyridine compounds (collismycin C and pyrisulfoxin A) from red alga-associated *Streptomyces* have been effective in reducing alveolar barrier damage caused by particulate matter-induced pulmonary injury (Choi et al., 2019). This is achieved by scavenging PM2.5-induced ROS, inhibiting the p38 MAPK pathway, and activating the PI3K/Akt pathway, thereby attenuating pulmonary microvascular endothelial permeability and leukocyte infiltration (Choi et al., 2019). These studies demonstrate that marine natural products effectively mitigate oxidative stress and inflammation by targeting both the NF-κB pathway and its interconnected signaling components, such as MAPK and PI3K/Akt, thereby highlighting their therapeutic potential for inflammatory lung diseases through a coordinated multi-pathway regulatory mechanism (Figure 3).

### 2.4 TLR4/MyD88

#### 2.4.1 The role of the TLR4/MyD88 pathway in lung injury

When lung tissue is exposed to pathogen-associated molecular patterns, such as bacterial lipopolysaccharide (LPS), which acts as an agonist for Toll-like receptor 4 (TLR4), LPS binds to TLR4. This binding then induces nuclear factor kappa-B (NF-κB) activity either through the Toll/interleukin-1 receptor (TIR)-domain-containing adaptor inducing interferon-β (TRIF) pathway or the myeloid differentiation primary response 88 (MyD88) pathway. Activated NF-κB triggers an inflammatory response by releasing pro-inflammatory cytokines, such as tumor necrosis factor-alpha (TNF-α), interleukin-1β (IL-1β), and interleukin-6 (IL-6). This, in turn, exacerbates pulmonary vascular permeability and alveolar epithelial injury (Doyle and O'Neill, 2006). In addition, the TLR4/MyD88 pathway can also activate the NLRP3 inflammasome, promoting the maturation and secretion of IL-8 and IL-1β, and further amplifying the inflammatory response (Guan et al., 2022). In terms of oxidative stress, this pathway increases the production of reactive oxygen species (ROS) by regulating the expression of NADPH oxidase (such as NOX4), leading to oxidative damage of lung tissue. Meanwhile, the excessive activation of the TLR4/MyD88 pathway can also induce apoptosis of alveolar epithelial cells and endothelial cells and disrupt lung barrier function by up-regulating the expression of pro-apoptotic proteins (such as Bax) and inhibiting the expression of anti-apoptotic proteins (such as Bcl-2) (Murphy and Caraher, 2015).

#### 2.4.2 Fucoxanthin regulates lung injury through the TLR4/MyD88 pathway

**FIGURE 4:**
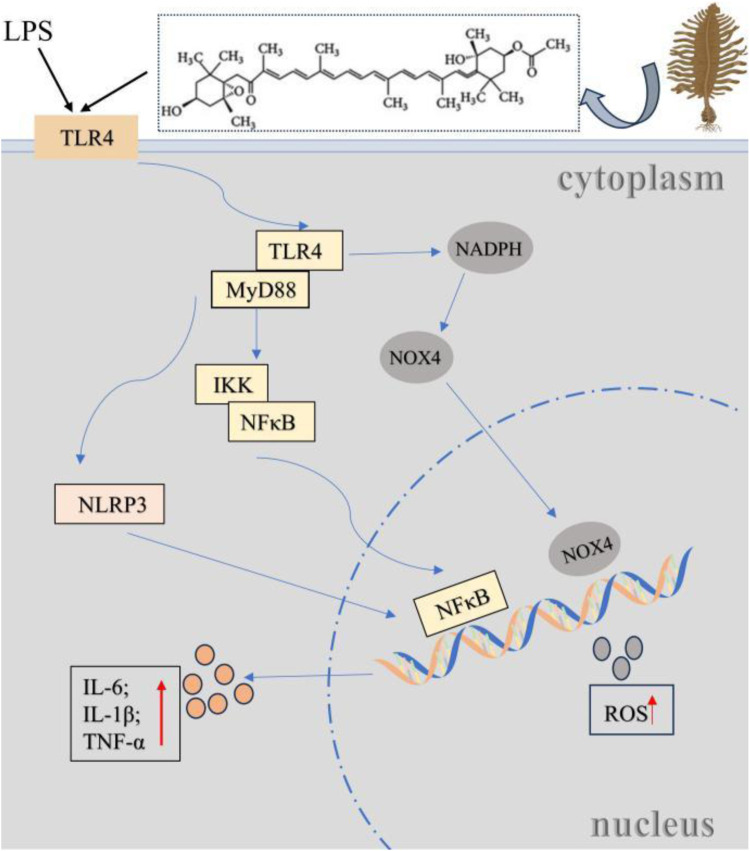
Fucoxanthin regulates lung injury through the TLR4/MyD88 pathway. Bacterial LPS (a TLR4 agonist) binds to the TLR4/MD-2 complex, activating NF-κB via MyD88 or TRIF pathways to release pro-inflammatory cytokines (e.g., TNF-α, IL-1β, IL-6), and triggering NLRP3 inflammasome activation, ROS production and apoptosis, thereby inducing acute lung injury (ALI) through inflammation, oxidative stress, and lung barrier disruption. Marine fucoxanthin inhibits LPS-induced ALI by competitively binding to the TLR4/MD-2 hydrophobic pocket, blocking TLR4 dimerization and MyD88-dependent signaling.

Marine fucoxanthin can significantly inhibit LPS-induced acute lung injury (Li et al., 2020). The core mechanism lies in directly binding to the hydrophobic pocket of the TLR4/MD-2 complex (partially overlapping with the LPS binding site). Molecular conjugating confirmed that this binding depends on the hydrogen bonds and hydrophobic interactions of key amino acids such as LYS-360 and PHE-151, thereby competitive blocking of TLR4 dimerization (Figure 4). This effect effectively inhibits downstream MyD88-dependent signal transduction, reduces the phosphorylation of the NF-κBp65 subunit and blocks its nuclear translocation, and ultimately significantly downregulates the expression of inflammatory mediators such as COX-2 and iNOS, as well as pro-inflammatory factors such as IL-6 and TNF-α. This study confirmed that fucoxanthin regulates the inflammatory storm through the ‘TLR4-MyD88-NF-κB axis’, providing a novel Marine drug candidate strategy for the treatment of acute lung injury.

### 2.5 Matrix Metalloproteinase 9 and fibroblasts

#### 2.5.1 Matrix Metalloproteinase 9 and fibroblasts

The development of pulmonary fibrosis involves complex molecular network regulation, among which the abnormal activation of the Matrix Metalloproteinase family plays a key role. Matrix metalloproteinases (MMP) are a zinc-dependent endoprotease family that are involved in the degradation and remodeling of extracellular matrix (Cui et al., 2017). In MMP, MMP-9 is particularly worthy of attention because it is elevated in patients with acute lung injury and acute respiratory distress syndrome, and is positively correlated with the severity of lung injury (Lanchou et al., 2003; Hsu et al., 2015; Ricou et al., 1996). The regulatory role of MMP-9 in lung injury shows significant complexity. On the one hand, its excessive activation directly aggravates pulmonary edema and increased permeability by degrading key extracellular matrix (ECM) proteins such as type IV collagen (Torii et al., 1997), the main components of the basement membrane, and destroying the integrity of the alveolar-capillary barrier. Meanwhile, MMP-9 amplifies the inflammatory cascade reaction by cutting and activating multiple pro-inflammatory factors and chemokines, promoting the recruitment and activation of inflammatory cells such as neutrophils into lung tissue, and causing tissue damage (Takahashi et al., 2018). On the other hand, the activity of MMP-9 also profoundly affects the process of tissue repair and remodeling. In the later stage of injury, the continuously high expression of MMP-9 not only hinders the normal reconstruction of ECM, but also the fragments produced by its degradation of ECM and the released/activated latent growth factors may instead promote the proliferation, migration and transformation into myofibroblasts of fibroblasts, driving the abnormal pathological fibrosis process (Chapman et al., 2004). Therefore, MMP-9 plays a crucial double-edged sword role in lung injury: early overactivation exacerbates acute inflammatory damage and barrier destruction, while persistent dysregulation in the later stage becomes an important molecule promoting the transformation from inflammation to fibrosis.

#### 2.5.2 Hirsutanol A and fucoidan alleviate lung injury through the regulation of matrix metalloproteinase-9 (MMP-9) and fibroblast function

**FIGURE 5:**
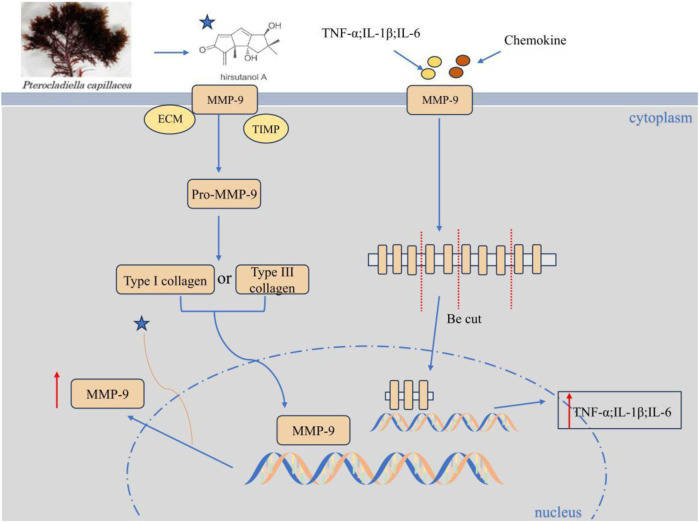
Hirsutanol A inhibits the expression of MMP-9. HA concentration-dependently suppresses LPS-triggered MMP-9-mediated gelatinolysis and its protein/mRNA expression (without affecting TIMP-1 activity), inhibits pro-MMP-9 induction, and significantly attenuates TNF-α, IL-6, and IL-1β levels.

Animal experiments have confirmed that in acute lung injury complicated with LPS-induced endotoxemia, Hirsutanol A (HA) derived from the Marine fungus Chondrostereum sp. NTOU4196 can alleviate lung tissue injury conditions (such as edema and inflammatory infiltration) (Jan et al., 2019). Meanwhile, the acute disease behavior is improved by inhibiting the activation of STAT3 and the expression of MMP-9 (Jan et al., 2019) (Figure 5). Another example is fucoidan from brown algae. It alleviates hyperoxia-induced lung injury in neonatal rats by regulating fibroblast differentiation, inhibiting the transformation of lung fibroblasts into myofibroblasts, manifested as a decrease in the expression of α-SMA and collagen I, reduced collagen deposition and pulmonary fibrosis (Zhang B. et al., 2020). HA and Fucoidan respectively alleviate lung tissue damage, fibrosis and oxidative stress by inhibiting the MMP-9-mediated inflammatory cascade and the transformation of fibroblasts into myofibroblasts, providing potential marine drug strategies for the treatment of lung injury.

### 2.6 αvβ3-FAK/Src pathway

#### 2.6.1 The role of the αvβ3-FAK/Src pathway in lung cancer

Cancer spread mainly occurs through the blood and lymphatic vessels. Many studies have shown that extracellular matrix proteins may be important in the formation of tumor blood vessels and lymphatic vessels (Wasik et al., 2022). In sepsis-induced lung injury, extracellular matrix proteins are abnormally activated, accelerating their binding to the integrin receptor αvβ3, thereby recruiting and phosphorylating focal adhesion kinase (FAK), activating downstream Src family kinases, and forming the FAK/Src signaling complex (Liu Y. S. et al., 2024; Lechertier and Hodivala-Dilke, 2012), Ultimately, it leads to an increase in endothelial permeability and disrupts the integrity of the endothelial barrier. In lung adenocarcinoma, von Willebrand factor (vWF), a multifunctional glycoprotein, inhibits angiogenesis by regulating angiopoietin-2 and integrin αvβ3 (Starke et al., 2011). Studies have shown that targeting and inhibiting αvβ3 or FAK/Src (such as the small molecule inhibitor PF-562271) may be one of the effective ways to slow down lung cancer (Wu et al., 2022).

#### 2.6.2 Isaridin E regulates lung injury complicated with sepsis through the αvβ3-FAK/Src pathway

**FIGURE 6:**
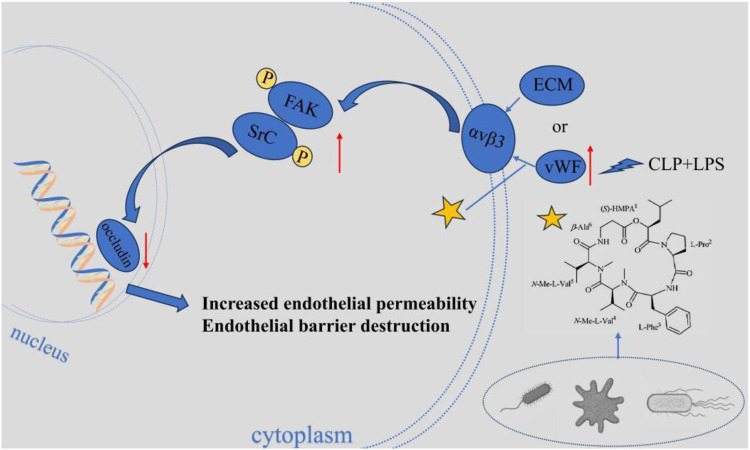
Isaridin E regulates lung injury complicated with sepsis through the αvβ3-FAK/Src pathway. Isaridin E dose-dependently curtails vWF release from activated platelets, inhibits platelet adhesion to LPS-stimulated HUVECs to reduce vWF secretion, and blocks vWF binding to integrin αvβ3 to inhibit downstream αvβ3-FAK/Src pathway activation. These actions collectively mitigate endothelial hyperpermeability, lung injury.

Isaridin E (ISE) is a marine-derived fungal cyclohexadepsipeptide. It can protect against sepsis through the αvβ3-FAK/Src pathway (Liu Y. S. et al., 2024). In sepsis, the release of vWF from platelets and damaged endothelium significantly increases. After vWF binds to the integrin receptor αvβ3 on the surface of endothelial cells, it activates the downstream focal adhesion kinase (FAK) and Src tyrosine kinase, triggering phosphorylation at the FAK (Y397) and Src (Tyr416) sites (Liu Y. S. et al., 2024). The abnormal activation of this pathway leads to the downregulation of endothelial cell junction proteins (such as VE-cadherin and occludin) expression, disrupts the integrity of the endothelial barrier, increases pulmonary vascular permeability, and triggers pulmonary edema and acute lung injury. ISE blocks this pathological process through dual actions (Figure 6). On one hand, ISE inhibits platelet activation and endothelial injury, reducing the release of vWF; On the other, ISE directly downregulates the expression of αvβ3 receptors in endothelial cells, blocking the binding of vWF to αvβ3, thereby inhibiting the phosphorylation activation of the FAK/Src pathway. Ultimately, ISE maintains endothelial barrier function by inhibiting the αvβ3-FAK/Src signaling axis, thereby alleviating pulmonary vascular leakage and tissue damage.

### 2.7 SP1 pathway

#### 2.7.1 The importance of SP1 in lung cancer

Sp1 is a founding member of the zinc finger transcription factor family (Black et al., 2001). It is famous for its ability to regulate gene expression in many biological processes. It also plays a key regulatory role in the development of lung cancer. It activates or inhibits the transcription of a series of genes involved in the malignant process of tumors by binding to the GC-box in the promoter region of the target gene (Li and Davie, 2010). Studies have demonstrated that Sp1 is overexpressed in various tumor diseases (Vizcaíno et al., 2015). Sp1 can promote cells to enter the S phase and drive the proliferation of tumor cells by activating cyclin, proto-oncogene MYC and growth factor receptor IGF1R. In terms of apoptosis, overexpression of Sp1 can activate anti-apoptotic genes and members of the Bcl-2 family, thereby promoting the survival of tumor cells (O'Connor et al., 2016). The expression level and activity of SP1 are often abnormally elevated in lung cancer tissues, and its high expression is significantly associated with a poor prognosis (Beishline and Azizkhan-Clifford, 2015), To make it a potential therapeutic target, inhibiting SP1 or its downstream pathways has demonstrated anti-tumor effects in preclinical studies.

#### 2.7.2 Naamidine J inhibits the nuclear translocation of SP1 by targeting CSE1L to slow down lung injury

**FIGURE 7:**
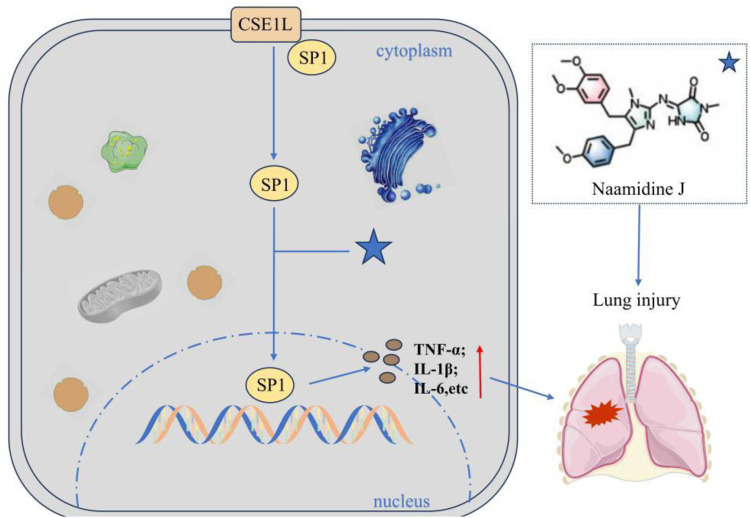
Naamidine J inhibits the nuclear translocation of SP1. Naamidine can directly bind to CSE1L’s His745 and Phe903 sites to block CSE1L-mediated nuclear translocation of the transcription factor SP1, thereby down-regulating pro-inflammatory factors (e.g., TNF-α, IL-1β, IL-6) and up-regulating anti-inflammatory factors.

Naamidine J (NJ) is a marine alkaloid and has been identified as a specific inhibitor of the nuclear transport protein CSE1L through chemical proteomics technology. Studies have confirmed that NJ directly binds to the His745 and Phe903 sites of CSE1L, blocking the nuclear translocation process of the transcription factor SP1 mediated by them (Gao et al., 2024). In the LPS-induced macrophage inflammation model, NJ significantly reduces the transcriptional activity of SP1 by inhibiting its nuclear entry, thereby down-regulating the expression of pro-inflammatory factors (TNF-α, IL-1β, IL-6, etc.) and up-regulating anti-inflammatory factors (Figure 7). This study for the first time reveals that the CSE1L-SP1 axis can serve as a therapeutic target for acute lung injury and provides a lead compound for the development of anti-inflammatory drugs targeting CSE1L.

## 3 Discussion

Marine natural products, derived from marine plants, animals, and microorganisms, represent a highly promising and efficient source of pharmaceuticals, exhibiting significant application potential in the field of lung injury treatment. This review systematically summarizes six key mechanistic pathways. Studies have demonstrated that by regulating core signaling pathways such as αvβ3-FAK/Src, TLR4/MyD88, NF-κB, and Keap1-Nrf2/HO-1/STAT3, marine natural products can effectively inhibit the release of inflammatory factors, alleviate oxidative stress responses (Li et al., 2020; Lee et al., 2025; Choi et al., 2019; Jan et al., 2019).

In terms of immunoinflammatory regulation, a variety of marine natural products exert their effects by targeting key pathways in different models, thereby inhibiting inflammatory responses and alleviating lung injury. In the pathways associated with oxidative stress and cell protection, marine-derived carotenoids exhibit prominent performance. They can regulate relevant signaling pathways and metabolic processes, improve the integrity of the alveolar barrier, and mitigate ferroptosis-related damage. Furthermore, as the core of inflammatory regulation, NF-κB-related pathways are synergistically targeted by multiple marine natural products, which inhibit the activation of related pathways. This further confirms the potential of marine natural products in alleviating inflammatory lung injury.

To date, more than 20 marine-derived drugs have been used in clinical practice. Most of the approved marine compounds are indicated for anti-tumor therapy, with a few being applied in the treatment of chronic pain, neuroinflammation, and bacterial infections (Wang et al., 2022), as well as serving as omega-3 fatty acid supplements in diets (Haque et al., 2022). However, drugs specifically for lung injury treatment remain scarce. Existing studies have shown that marine natural products such as astaxanthin, fucoxanthin, brown algal polysaccharides, and coral extracts exhibit high safety profiles and convenient administration routes. Moreover, their multi-target regulatory properties are highly compatible with the complex pathological processes of lung injury, providing new insights and candidate drugs for lung injury treatment and demonstrating certain prospects for clinical translation.

In the future, further in-depth studies on *in vivo* mechanisms of action should be conducted, and multi-omics technologies can be employed to dissect the panoramic regulatory networks of marine natural products (Leão et al., 2021). In terms of experimental models, it is necessary to establish more clinically relevant composite models and carry out cross-species validation studies to reduce the differences in drug responses between animals and humans. For clinical applications, systematic evaluations of the pharmacokinetic and toxicological properties of marine natural products are required to achieve the synergistic effects of multiple pathways *in vivo*. Additionally, efforts should be made to develop novel formulations to improve their bioavailability and targeting, while exploring insufficiently studied marine biological resources such as marine microorganisms and symbiotic algae, thereby providing a broader scope for the development of therapeutic drugs for lung injury.
